# Macropinocytic dextran facilitates KRAS-targeted delivery while reducing drug-induced tumor immunity depletion in pancreatic cancer

**DOI:** 10.7150/thno.65299

**Published:** 2022-01-01

**Authors:** Fang Yuan, Mengnan Sun, Zhengsheng Liu, Huiqin Liu, Weijian Kong, Rui Wang, Feng Qian

**Affiliations:** School of Pharmaceutical Sciences, Beijing Advanced Innovation Center for Structural Biology, and Key Laboratory of Bioorganic Phosphorus Chemistry & Chemical Biology (Ministry of Education), Tsinghua University, Beijing 100084, China.

**Keywords:** Pancreatic cancer, targeted drug delivery, KRAS mutation, dextran, tumor microenvironment

## Abstract

**Background**: Pancreatic cancer comprises not only cancer cells but also a collection of cross-talking noncancerous cells within tumor. Therefore, selective delivery of cytotoxic agents towards cancer cells and limiting the collateral damage to tumor suppressive benign cells, such as effector lymphocytes in the tumor microenvironment, is of great value.

**Methods**: Pancreatic cancer cells harbor oncogenic KRAS which induces a constitutively high level of macropinocytosis. Inspired by such uniquity, we sought to explore the targeting potential of dextran, a biomaterial presumed to be endocytosed in the macropinocytosis dependent manner. Cell entry preference, mechanism and subcellular sorting of dextran with different molecular weights were firstly examined. Triptolide (TP), a potent cytotoxin was then set as the model payload for dextran conjugation. KRAS selectivity and the therapeutic effects of dextran-conjugated TP were investigated via both *in vitro* cellular studies and *in vivo* tumor model assessment.

**Results**: Dextran, with a specific molecular weight of 70 kDa rather than other weights, was identified as a robust KRAS-responsive intracellular delivery carrier with enhanced entry upon KRAS mutation. The 70 kDa dextran-conjugated TP (DEX-TP) displayed greater efficacy and cellular deposition efficiency towards KRAS mutant cells than KRAS wild-type cells. Treatment with DEX-TP suppressed tumor progression in KRAS mutant pancreatic cancer orthotopic mouse models with reduced toxicity and significantly extended mouse survival time. Furthermore, the conjugate attained a more favorable therapeutic outcome in the tumor immune microenvironment than the free drug, preserving the fraction of T cells and their effector cytokines.

**Conclusions**: In summary, macropinocytic dextran was able to provide drug delivery selectivity towards KRAS mutant cancer cells and reduce tumor immunity depletion caused by the cytotoxic drug in pancreatic cancer.

## Introduction

Cancer cells, which are the progenitors of tumor initiation and progression, are undoubtedly the central target of oncological medicines. Targeted inhibition of such cells based on their specific genetic mutation has been consistently pursued, yet not all cancer cells are targetable. With >90% of patients harboring the “undruggable” KRAS mutant alleles (i.e. KRAS^G12D^, KRAS^G12V^, and KRAS^G12R^), pancreatic ductal adenocarcinoma (PDAC) is notoriously lethal with very limited molecular targeting therapeutic options 1.

On the other hand, some compounds that are extremely potent against PDAC cancer cells are associated with low selectivity and high off-target toxicity. Triptolide (TP), a natural product derived from *Tripterygium wilfordii Hook F.*, is one such compound. TP has been able to restrain PDAC progression effectively in a series of preclinical models, and achieved a higher tumor response rate than gemcitabine (GEM) and 5-FU 2. Translation of TP has nevertheless been impaired due to its systemic toxicity after administration 3. Moreover, accompanying the recognition of the therapeutic value of antitumor immunity, a growing concern for TP use is its severe immunosuppressive activity. TP induces the elimination and functional inhibition of T cells 4; however the existence and activation of T cells within the TME correlate positively with the prognosis of advanced pancreatic cancer 5. Therefore, a drug delivery strategy may be desired to selectively deliver these compounds into PDAC cancer cells, which are generally KRAS mutant, and avoid collateral damage to unwanted cellular components, such as lymphocytes in the TME.

Macropinocytosis is a nonabsorptive endocytic process induced by plasma membrane ruffling and actin polymerization 6. Since it was first observed in 1931, this process has been considered highly conserved among eukaryotic cells internalizing extracellular macromolecules, such as proteins, lipids and viruses 6. Oncogenic RAS was found to regulate the activity of macropinocytosis; the introduction of either RAS oncogenes or RAS proteins induces such a scavenging pathway directly 7,8. Functional contributions of this phenotype were recently further identified in KRAS mutant PDAC progression; that is, enhanced macropinocytosis of extracellular fluids serves as a rapid amino acid supply route for pancreatic cancer cells^9^,^10^. This metabolic feature is retained across cancer cells from different PDAC subtypes and can be enhanced by nutrient deprivation 11.

Dextran, a natural polysaccharide composed of D-glucose, is a biomaterial that is widely used in medical products and drug formulations 12. Due to its high hydrophilicity, low immunogenicity and great chemical feasibility, dextran has long been applied as a polymeric carrier in prodrug conjugation to improve drug solubility, reduce toxicity, and optimize systemic pharmacokinetics 12,13. Here, we intend to assess a previously unexplored drug delivery capability of dextran based on its cellular entry habit; dextran is a commonly used macropinocytosis probe that enters cells accompanying membrane ruffling and macropinocytic vesicle formation 14. Indeed, since the 1980s, dextran and its soluble derivatives have long been used by biologists to evaluate the level of cell pinocytosis activity 15. Linking this preference to the aforementioned KRAS transformation discovery, we envisioned that dextran might offer unique KRAS selectivity and thus would be desirable for PDAC cancer cell targeted drug delivery.

In this work, we discuss the rational design of KRAS mutant cancer cell targeting dextran-conjugated drug and illustrate its benefits. Dextran with different molecular weights could correspond to different cellular interaction modes, especially in terms of their endocytic preference 16,17. Therefore, the proper choice of a KRAS-responsive dextran was first examined, touching on the debatable perception that the macropinocytosis dependency of macromolecules could vary by size. The KRAS selectivity of the drug conjugate using dextran with the right size was then assessed, and any favorable *in vivo* effects, in particular, relief of the tumor immune microenvironment, were studied using multiple complementary pancreatic tumor mouse models. Collectively, we present an alternative strategy to target KRAS-mutant PDAC cancer cells using dextran polymeric conjugation.

## Materials and Methods

### Cell Lines and Animals

Cell lines were obtained from either the American Type Culture Collection (MIA PaCa-2, Panc-1, HEK293, and Raji) or the Cell Resource Center of Peking Union Medical College (BxPC-3, NIH3T3, and Jurkat) and regularly maintained in the lab. Culture was based on either DMEM (MIA PaCa-2, Panc-1, HEK293, and NIH3T3) or RPMI 1640 (BxPC-3, Jurkat, and Raji) supplemented with 10% fetal bovine serum (Gemini, Australia) and 1% penicillin and streptomycin (Life Technologies, USA) under 5% CO_2_ at 37°C.

BALB/c nude mice and C57BL/6 mice used for tumor model establishment were ordered from Beijing Vital River Laboratory Animal Technology and monitored regularly in Laboratory Animal Research Center of Tsinghua University. Animal care was under surveillance during the experiments, and the protocols were approved by the Institutional Animal Care and Use Committee of Tsinghua University and carried out in accordance with the People's Republic of China Legislation Regarding the Use and Care of Laboratory Animals.

### Materials

Reagents were purchased from commercial sources: FITC-labeled 4 kDa, 70 kDa, 150 kDa dextrans (Sigma, USA), TMR 70 kDa-labeled dextran (Life Technologies), TP (Chengdu Purifa, China), succinic anhydride (J&K Scientific, China), DMAP (Macklin, China), CDI (Macklin, China), esterase from porcine liver (Sigma, USA), EIPA (MedChemExpress, USA), and LysoTracker (Life Technologies).

Plasmids were obtained from Addgene (USA): pBABE-Puro-KRAS^G12V^ was a gift from Christopher Counter and the pBABE-Puro vehicle control was a gift from Hartmut Land, Jay Morgenstern and Bob Weinberg. All plasmids were packaged into lentiviruses with RSV, VSV, and pMDL and transduced into BxPC-3 cells via infection and selected under puromycin pressure (~1.5 µg/ml).

Antibodies used for immunohistochemical staining were from Servicebio (China): anti-Ki67 (GB111141), anti-EpCAM (GB11274), anti-CD3 (GB13014), anti-CD4 (GB13064-2), anti-CD8 (GB13429), anti-CD45 (GB11066), anti-IFN-γ (GB11107-1) and anti-TNF-α (GB11188).

### Cellular Entry Mechanism and Selectivity

Identification of the macropinocytic entry of extracellular particles was based on the change of the particle cellular entry level after EIPA treatment. EIPA is a highly specific inhibitor of macropinocytosis, and the entry of macropinocytic substrates is disturbed by EIPA administration 18. A total of 100,000 cells were first seeded in 12-well plates and treated with or without 50 μM EIPA for 30 min. FITC-labeled dextrans of different sizes were then added for another 30 min and cells were later collected for subsequent single-cell analysis using a flow cytometer (FITC florescence detection, BD LSRFortessa, BD Bioscience).

Investigation of the cellular entry selectivity of dextran was performed by comparing the level of cellular entry between KRAS mutant cells and KRAS wild-type cells, and both flow cytometry and confocal laser scanning microscopy (CLSM) were used for cross-checking. For flow cytometer-aided experiments, 100,000 cells were seeded in 12-well plates, and FITC-labeled dextrans of different sizes were added the next morning. Different concentrations (1.0 mg/ml and 0.1 mg/ml) and incubation periods (3-48 h) were examined, and at each experimental time point, cells were rinsed with PBS and sent before cytometric analysis. For CLSM-aided experiments, cells were seeded in glass bottom cell culture dishes at 50% confluency. FITC-labeled dextran (1 mg/ml) was used for comparison between MIA PaCa-2 and BxPC-3 cells, while for experiments involving BxPC-3 KRAS^G12V^-overexpressing cells, TMR-labeled dextran was exploited instead due to the self-containing GFP marker within the plasmids, and signals are presented as pseudocolored green. Microscopy images were captured at 60× magnification with an A1 confocal microscope (Nikon) and analyzed with NIS-Element 5.2 software (Nikon). Colocalization coefficients were generated automatically, while dextran indexes were calculated based on a previously reported method 19, which in general computed the area fraction of endocytosed dextran.

### Construction and Purification of DEX-TP

DEX-TP was constructed through the conjugation of TP succinate to 70 kDa dextran. Triptolide succinate (0.2 mM), whose preparation was described previously^20^, was dissolved in 3 ml of DMSO together with 0.35 mM CDI and stirred for 1 h under nitrogen protection. Then, 300 mg of 70 kDa dextran was dissolved in 6 ml of DMSO and added together with 0.5 ml of TEA into the previous 3 ml reaction system, and the resulting mixture was further stirred for 24 h at room temperature. After the reaction, ethanol:diethyl ether (1:1) was added for purification, where the white residue was collected and subjected to washing with methanol. The crude product after washing was later dried in a vacuum oven and again purified by size exclusion chromatography (Desalted Superdex 200, GE Healthcare) for the final product.

### TP Content Determination and Drug Release Study of DEX-TP

The content of TP within the drug conjugates was determined by measuring the released TP and TP succinate after the basic hydrolysis of the drug conjugates. Briefly, 5 mg of DEX-TP was dissolved in 0.2 ml of water and then added to 0.2 ml of 0.1 M NaOH. The solution was fully mixed by vortexing and then left out overnight at room temperature for ester hydrolysis. The next morning, 0.2 ml of 0.1 M HCl was added, and then 0.1 ml of this solution was sampled and mixed further with 0.9 ml of methanol. The supernatant after methanol addition and centrifugation at 12,000 rpm for 10 min was subjected to HPLC for TP concentration determination.

The release kinetics of TP contents from the drug conjugates in 10 U/ml esterase containing medium or esterase-free medium were studied to understand drug stability and cleavage. 10 mg of DEX-TP was dissolved in 2 ml of release media, and at each experimental time point, a 0.1 ml sample was withdrawn and mixed with 0.9 ml of methanol. This solution was then centrifuged at 12,000 rpm for 10 min, and the supernatant was subjected to HPLC analysis.

The HPLC method for TP quantitative analysis used a C18 column with a mobile phase of water:acetonitrile (25:75) at a flow rate of 1 ml/min and UV detection at 218 nm.

### *In vitro* Drug Efficacy Study

Dose-response curves of TP and DEX-TP across multiple cell lines were plotted to evaluate drug efficacy and cellular deposition efficiency. Generally, cells were seeded at a density of 1,000-3,000 cells per well in 96-well plates and underwent drug titration once attached. Drug treatments lasted 72 h, and doses ranged from 1 nM to 100 μM. After incubation, the cells were gently aspirated with fresh media for further CellTiter Glo (Promega)-mediated cell viability assays. Readouts were fitted to drug response curves, and the corresponding IC_50_ values were determined with GraphPad Prism software (version 6.0).

### Subcutaneous Tumor Models

To evaluate the antitumor effects of DEX-TP in tumor models with different KRAS gene backgrounds, MIA PaCa-2-, BxPC-3-, and BxPC-3 KRAS^G12V^-derived subcutaneous xenograft models were established using immunodeficient BALB/c nude mice. For subcutaneous xenografts, no more than 0.1 ml of cancer cell-containing liquids (approximately 200,000 cells) was injected into the right rear flank area of each nude mouse. The tumor volume was calculated as (length×width×width/2), and when the volume was approximately 100 mm^3^, treatments started. The mice were randomly grouped and administered saline, TP (0.3 mg/kg) or DEX-TP (1 mg/kg, an equivalent dose of TP) every two days for a total of seven treatments via tail vein injection.

### Orthotopic Tumor Models

Two types of orthotopic pancreatic tumor models were exploited in this study: a human pancreatic cancer cell line-derived xenograft model and a KPC mouse tumor tissue-derived allograft model. For xenografts, approximately 500,000 MIA PaCa-2-luciferase cells were injected into the head of the pancreas of each immunodeficient BALB/c nude mouse under anesthesia. One to two weeks was generally needed for tumor establishment. Mice were then randomly grouped, and treatments started with saline, TP (0.3 mg/kg), DEX-TP (0.3 mg/kg, an equivalent dose of TP), DEX-TP (1 mg/kg, an equivalent dose of TP), or GEM (30 mg/kg) every two days for a total of eight treatments via tail vein injection. The growth and progression of the tumors were monitored with bioluminescence imaging after intraperitoneal injection of 150 mg/kg luciferin. The mice were then anaesthetized with 0.3 mL Avertin and 7-10 min later put into the imaging cube for bioluminescence image acquirement, using an IVIS Lumina II (Caliper, exposure time: 30 s) and analyzed for their average radiance with Living Image software (Caliper). For allografts, precut KPC mouse tumor tissues (diameter of approximately 3 mm) were transplanted into the head area of the pancreas of each immunocompetent C57BL/6 mouse under anesthesia. Mice were randomized, and drug dosing started 1 week after surgery. Since the main purpose of this study was to compare the drug effects of DEX-TP and free TP in a clinically relevant model, drugs were administered weekly at the same dose (0.3 mg/kg, an equivalent dose of TP).

### Histological Analysis

For histological analysis, tumor-bearing mice were randomly selected and sacrificed at the end of the experiments. Tissues were collected and fixed in 10% formalin after paraffin embedding. Sections (approximately 4 μm) were conducted and stained according to the study purpose. Engaged antibodies: anti-Ki67 (GB111141, 1/500 dilution), anti-EpCAM (GB11274, 1/500 dilution), anti-CD3 (GB13014, 1/100 dilution), anti-CD4 (GB13064-2, 1/1000 dilution), anti-CD8 (GB13429, 1/400 dilution), anti-CD45 (GB11066, 1/1000 dilution), anti-IFN-γ (GB11107-1, 1/200 dilution) and anti-TNF-α (GB11188, 1/500 dilution).

Images were acquired by optical microscopy and analyzed by calculating the area fraction of positive signals per visual field with ImageJ (NIH), where for each section, three independent fields were randomly selected and compared under the same magnification.

### Statistical Analysis

Statistical analyses were conducted with GraphPad Prism software (version 6.0). For comparisons among two independent groups, unpaired two-tailed Student's t-tests were used. For comparisons among three or more independent groups, one-way ANOVA and multiple comparisons were performed. For survival analyses, Kaplan-Meier curves were plotted, and survival benefit comparisons were conducted using log-rank (Mantel-Cox) tests. P value format: ns p> 0.05, * p< 0.05, ** p<0.01, *** p<0.001, **** p<0.0001.

## Results

### Identification of 70 kDa Dextran as a KRAS-Responsive Carrier

**Figure 1:**
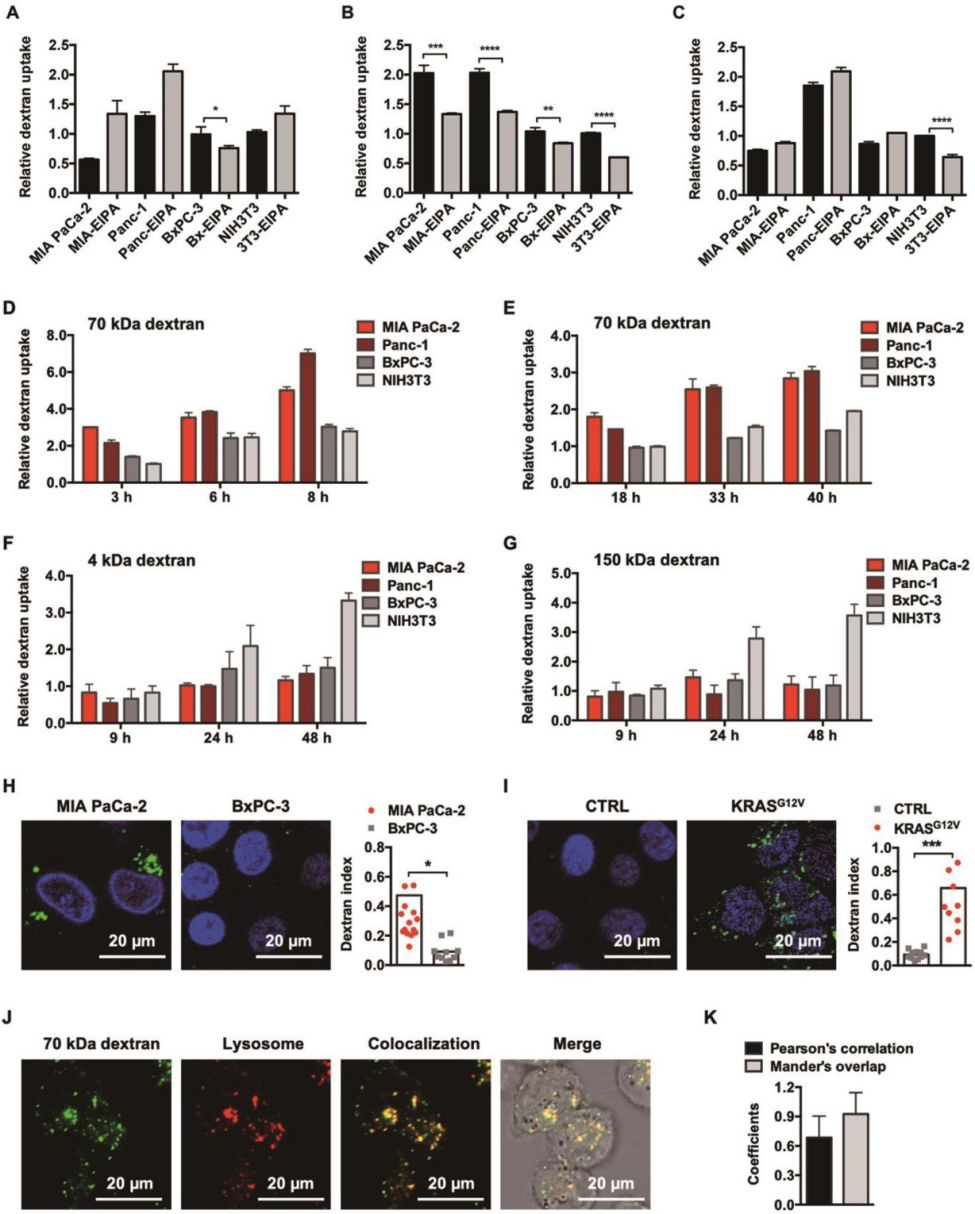
KRAS-responsive cell entry of 70 kDa dextran. (A, B and C) Comparison of 4 kDa (A), 70 kDa (B), and 150 kDa (C) dextran uptake with (gray) or without (black) 50 μM EIPA in multiple cell lines. Data are presented relative to the values obtained for NIH3T3 cells. Error bars indicate the mean and SD, n=3. (D, E, F and G) Comparison of 1.0 mg/ml 70 kDa (D), 0.1 mg/ml 70 kDa (E), 4 kDa (F), and 150 kDa (F) dextran uptake between KRAS mutant (reddish) and KRAS wild-type (grayish) cells by flow cytometry at different time points. Data are presented relative to the values obtained for NIH3T3 cells at the first time point. Error bars indicate the mean and SD, n=3. (H and I) CLSM-aided confirmation of the enhanced entry of 70 kDa dextran (green) towards KRAS mutant cells. Representative images and quantifications are presented: MIA PaCa-2 compared to BxPC-3 (H) and BxPC-3 (CTRL) compared to BxPC-3 KRAS^G12V^ (KRAS^G12V^) (I). (J) Lysosomal trafficking of 70 kDa dextran. Fluorescent signals: 70 kDa dextran (green), LysoTracker (red), colocalization of dextran and LysoTracker (yellow). (K) Pearson's correlation and Mander's overlap coefficients of the colocalization of 70 kDa dextran and LysoTracker. Error bars indicate the mean and SD, where at least 10 objects were observed.

We included 3 dextran candidates with varying sizes: 4 kDa, 70 kDa, and 150 kDa **(Figure S1)** at the beginning of this study. By examining their internalization under EIPA pressure, a common approach where the use of EIPA specifically impedes macropinocytosis^18^, we found that the robustness of macropinocytosis addiction among different sized dextrans was inconsistent. Among them, only dextran with a molecular weight of 70 kDa exhibited universal EIPA sensitivity across all tested cell lines, including two KRAS mutant cells (MIA PaCa-2 and Panc-1) and two KRAS wild-type cells (BxPC-3 and NIH3T3), demonstrating a robust macropinocytosis dependency** (Figure 1B)**. In contrast, though 4 kDa and 150 kDa dextran could be internalized via macropinocytosis into some cells (4 kDa dextran entered into BxPC-3 cells **(Figure 1A)** and 150 kDa dextran entered into NIH3T3 cells **(Figure 1C)**), they did not share the same level of dependency as 70 kDa dextran.

Accompanying this, we further observed that only 70 kDa dextran achieved a persistent, nonsaturable, enhanced cellular entry into KRAS mutant cancer cells over KRAS wild-type cells, as identified through a flow cytometer-aided long-term kinetic investigation **(Figure 1D and 1E)**, which was in clear contrast to the irregular pattern for the 4 kDa **(Figure 1F)** and 150 kDa **(Figure 1G)** dextrans. This enhancement was additionally confirmed by CLSM-aided experiments **(Figure 1H)** and suggested to be directly induced by KRAS mutation, since following the introduction of KRAS^G12V^ into BxPC-3 cells, the cellular uptake of 70 kDa dextran increased directly by 7-fold **(Figure 1I)**.

CLSM also confirmed that the post-endocytic event for 70 kDa dextran was lysosomal trafficking **(Figure 1J)** with little recycling out, as suggested by the colocalization analysis of intracellular dextrans and lysosomes **(Figure 1K)**. In addition, the cytotoxicity of 70 kDa dextran was examined, and no antiproliferative effects were observed, affirming its safety **(Figure S2)**. Therefore, we chose this specific molecular weight dextran for the following drug conjugate construction.

### Design and Characterization of Dextran-Conjugated Drugs

**Figure 2:**
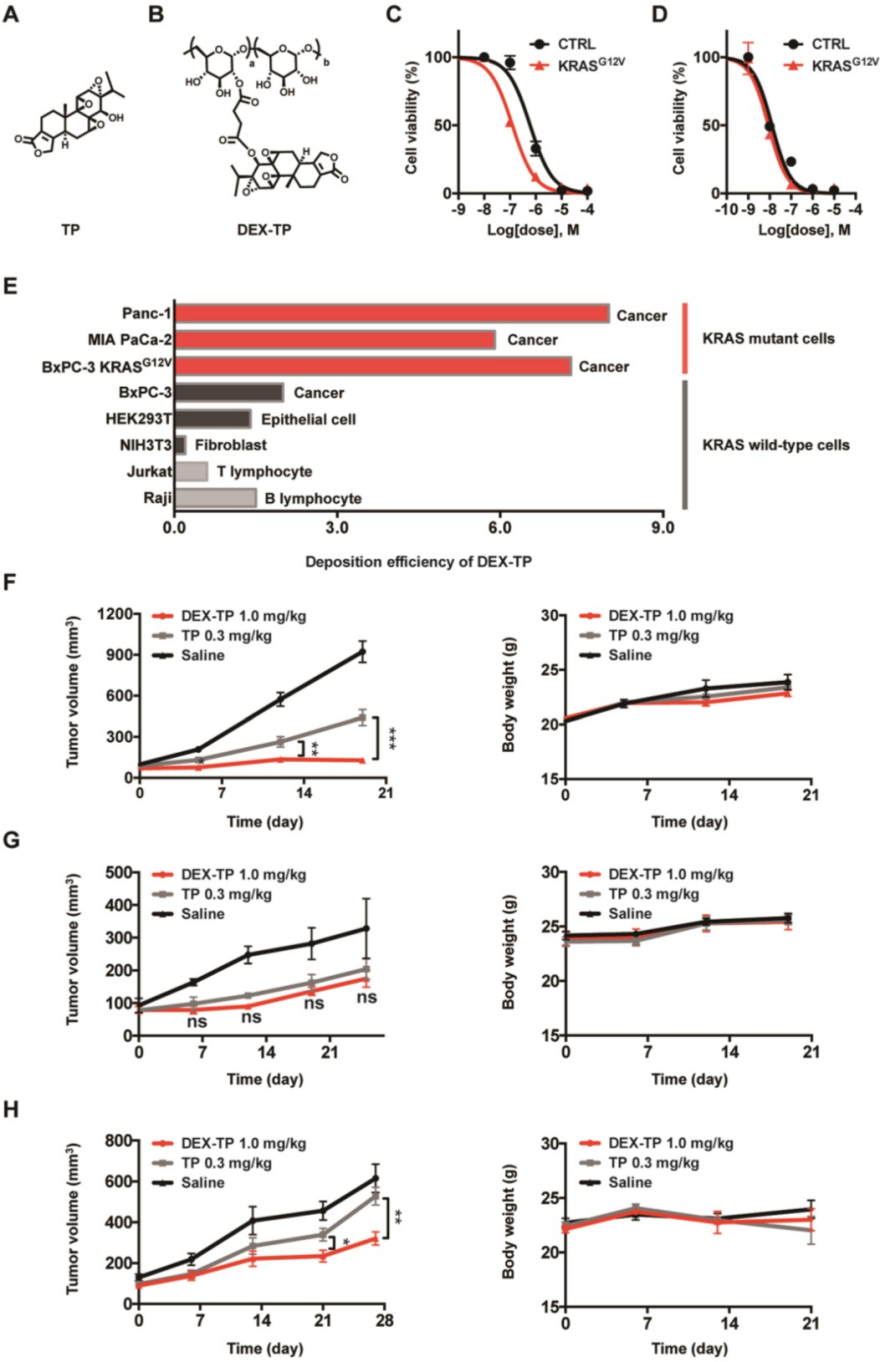
Enhanced efficacy, cellular deposition efficiency, and tumor growth inhibition of DEX-TP towards KRAS mutant cancer. (A and B) Chemical structures of TP (A) and DEX-TP (B). (C and D) Drug efficacy of DEX-TP (C) and TP (D) towards BxPC-3 (CTRL) and BxPC-3 KRAS^G12V^ (KRAS^G12V^) cells. Error bars indicate the mean and SD, n=3. (E) Cellular deposition efficiency of DEX-TP under different KRAS gene backgrounds. (F, G and H) Tumor growth curves (left) and body weight surveillance (right) under the following drug treatments: MIA PaCa-2-derived subcutaneous model (F), n (saline) = 7, n (TP) = 8, n (DEX-TP) = 8, and error bars indicate the mean and SD; BxPC-3 derived subcutaneous model (G), n (saline) = 4, n (TP) = 6, n (DEX-TP) = 6, and error bars indicate the mean and SD; BxPC-3 KRAS^G12V^-derived subcutaneous mode (H), n (saline) = 7, n (TP) = 7, n (DEX-TP) = 7, and error bars indicate the mean and SD. All treatments were given every two days for a total of 7 treatments via tail vein injection, and doses are denoted as TP equivalents.

Following the identification of 70 kDa dextran as a KRAS-responsive intracellular delivery carrier, we next tested the hypothesis that by using such a carrier, the targeted delivery of anticancer agents such as TP towards KRAS mutant cells could be possible. Integrating the literature 21 and our earlier experience, we conjugated TP **(Figure 2A)** with dextran through a succinate linker that coupled both parts through ester bonds **(Figure 2B)**. This design fit the confirmed subcellular transport of dextran, where succinate esters were once demonstrated to be cleavable by lysosomal enzymes 22. Removing the uncoupled TP succinate by size exclusion chromatography, we collected the DEX-TP product** (Figure S3A)**. The degree of TP labeling was 1.5% (TP:glucose unit, mole ratio), which represented 6-7 TP molecules on average for each 70 kDa dextran conjugated to the polymer chain. The conjugates were readily soluble in water, forming a clear solution, and exhibited a particle size comparable with fluorescently labeled dextran and native dextran, with no nanostructure formation or apparent surface charges** (Figure S3B)**. Moreover, the drug release of DEX-TP in esterase-containing medium was also studied. In clear contrast to the TP release in DMEM medium, with esterase addition, a responsive, sustained drug release profile appeared, indicating attainable intracellular cleavage** (Figure S3C)**.

### Enhanced Drug Delivery Selectivity towards KRAS Mutant Cancer Cells by Dextran Conjugation

DEX-TP exhibited cytotoxicity and antitumor effects both *in vitro* and *in vivo,* but its activity varied depending on the KRAS mutation status of the tested model. The effects of oncogenic KRAS on DEX-TP drug efficacy *in vitro* were first examined. By comparing the efficacy of free TP and DEX-TP on BxPC-3 and BxPC-3 KRAS^G12V^ cells, we found that DEX-TP displayed enhanced drug efficacy upon KRAS mutation **(Figure 2C)**, while free TP showed no selectivity **(Figure 2D)**.

Due to the kinetics of endocytic transport, dextran conjugation caused a decrease in drug potency, that is, an increase in drug IC_50_. We termed the “DEX-TP cellular deposition efficiency” as 
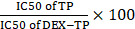
, to quantitively evaluate the efficiency of DEX-TP endocytic transport, where a higher deposition efficiency value indicated a more proficient endocytic transport. We found that there was an obvious gap as large as 3-10-fold in terms of DEX-TP cellular deposition efficiency between KRAS mutant cells and KRAS wild-type cells. For KRAS mutant cancer cells, their DEX-TP deposition efficiencies varied from 5.9 to 7.3; however, for KRAS wild-type cells, such values ranged from 0.2 to 2 **(Figure 2E and Figure S4)**.

We further investigated the impact of KRAS on DEX-TP *in vivo* efficacy. Subcutaneous xenograft models derived from MIA PaCa-2, BxPC-3 and BxPC-3 KRAS^G12V^ cells were established to compare the antitumor effects of DEX-TP and free TP in parallel, and in order to provide an equal comparison, both free TP and DEX-TP were given at their maximum tolerated doses (MTDs). In these head-to-head comparisons, we observed that in only KRAS mutant cell-derived xenograft models, that is, MIA PaCa-2- **(Figure 2F)** and BxPC-3 KRAS^G12V^- **(Figure 2H)** derived models, DEX-TP outperformed free TP with higher tumor growth inhibition rates. However, in stark contrast, such a difference did not exist in the BxPC-3-derived xenograft model, where it was observed that DEX-TP did not improve the therapeutic effects of the parent TP **(Figure 2G)**. However, once oncogenic KRAS^G12V^ was reintroduced, the therapeutic difference reappeared** (Figure 2H)**.

**Figure 3:**
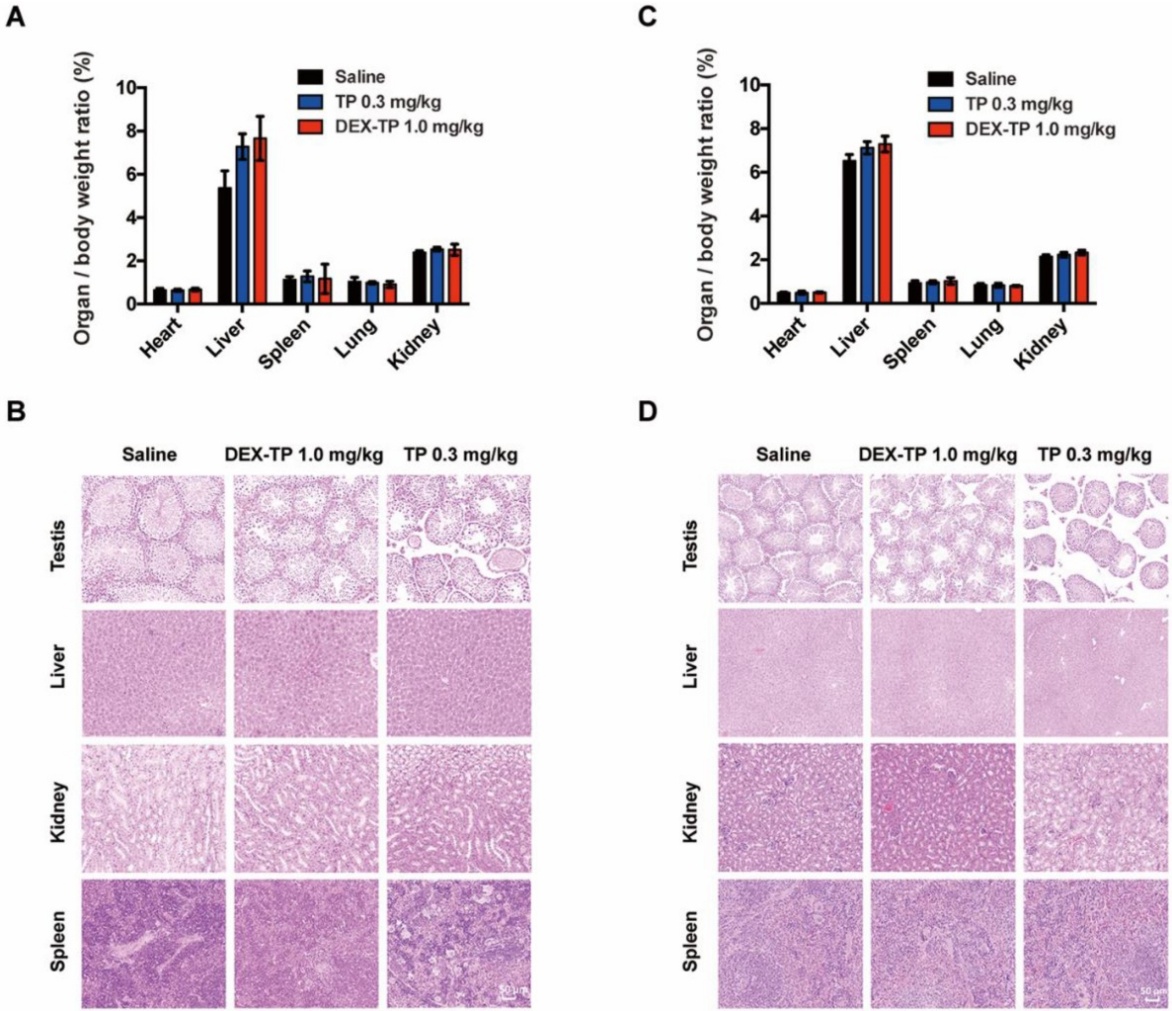
Safety evaluation of the DEX-TP treatments. (A and B) Assessment of the organ/body weight ratio (A) and HE staining of key organs (B) after drug treatment in the MIA PaCa-2-derived subcutaneous tumor model. (C and D) Assessment of the organ/body weight ratio (C) and HE staining of key organs (D) after drug treatment in the BxPC-3-derived subcutaneous tumor model. Error bars indicate the mean and SD from at least 3 independent experiments.

In addition to its selectivity towards KRAS mutant cells, DEX-TP was also well tolerated. Dextran conjugation was able to deliver more than 3-fold higher TP (1.0 mg/kg, equal dose of TP) with good tolerability, without incurring severe adverse effects, such as drug-induced animal death or significant body weight loss **(Figure 2F, 2G and 2H)**. We noticed an increasing of liver/body weight ratio under both DEX-TP and TP treatments **(Figure 3A and 3C)**, which might suggest tissue injury. However, no significant histological difference was observed among all three groups **(Figure 3B and 3D).** Besides, TP is a compound with severe reproductive toxicity; however, using DEX-TP treatment, even a relatively higher dose did not confer the same extent of toxicity as free TP with a much lower dose **(Figure 3B and 3D)**.

### DEX-TP Restrained Orthotopic Pancreatic Tumor Progression

**Figure 4:**
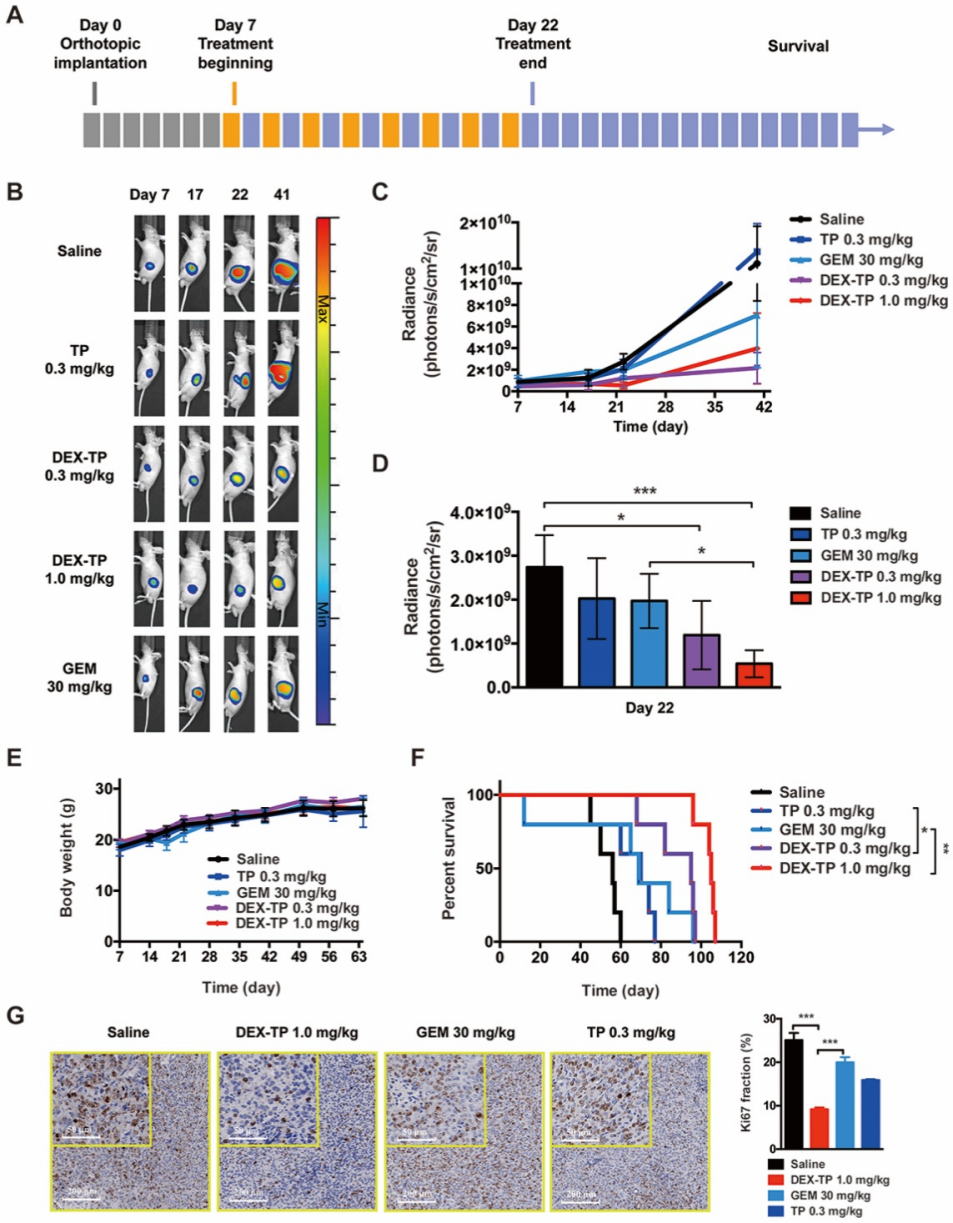
DEX-TP restrained orthotopic MIA PaCa-2 tumor growth. (A) Scheme of experiment. Treatments were given every two days for a total of 8 treatments via tail vein injection, with n=5 for all groups. Doses are denoted in the figure, while DEX-TP is indicated as the equivalent dose of TP. (B) Representative bioluminescence images of anesthetized mice from al treatment groups before (day 7, left) during (day 17, middle left), immediately after 8 doses (day 22, middle right) and 20 days after dosing (day 41, right). (C) Overview of the orthotopic tumor growth curve from the qualitative analysis of all luminescence images. Error bars indicate the mean and SD. (D) Qualitative analysis of the luminescence intensity at day 22, when the dosing ended. Error bars indicate the mean and SD. (E) Body weight surveillance during experiments. Error bars indicate the mean and SD. (F) Kaplan-Meier survival curves of orthotopic MIA PaCa-2 tumor-bearing mice. (G) Ki67 analysis of tumor sections from each treatment group. Representative staining pictures (left) and quantification (right). Error bars indicate the mean and SD, n=3.

Mice with luciferase-expressing orthotopic MIA PaCa-2 tumors, a model with a distinct physiological environment and disease progression in comparison to a subcutaneous model, were prepared to advance the therapeutic evaluation of DEX-TP. Tumor growth (monitored by bioluminescence) and mouse survival were documented, and remedies including DEX-TP, free TP, and PDAC first-line chemotherapy drug GEM were compared simultaneously **(Figure 4A)**.

Basing on the luminescence images, both 0.3 mg/kg and 1.0 mg/kg DEX-TP significantly inhibited tumor growth throughout the time course of the experiments **(Figure 4B and 4C)**, and at day 22, when treatment ended, 1.0 mg/kg DEX-TP clearly displayed antitumor superiority over GEM **(Figure 4D)**. During treatment, there were no observed safety issues or body weight losses from DEX-TP treatments **(Figure 4E)**; however, for the GEM and free TP treatment groups, drug-related mouse deaths occurred in each group. Survival benefits **(Figure 4F)** and proof of enhanced cancer cell killing **(Figure 4G)** over free drug under DEX-TP treatment, as a consequence of the above items, were revealed with statistical significance.

### DEX-TP Prolonged the Survival of KPC Allograft Mice and Alleviated Immune Depletion by TP

We previously mentioned that one of TP's undesirable effects is leukocyte suppression. In fact, TP, as an immunosuppressor, has been applied to treat autoimmune diseases for hundreds of years in traditional Chinese medicine 23. However, cancer therapy is trending to leverage the immune system to oppose tumor progression. Since dextran was demonstrated to improve the cancer cell selectivity of TP, we wondered whether these conjugates would behave in a more immune system-friendly manner.

**Figure 5:**
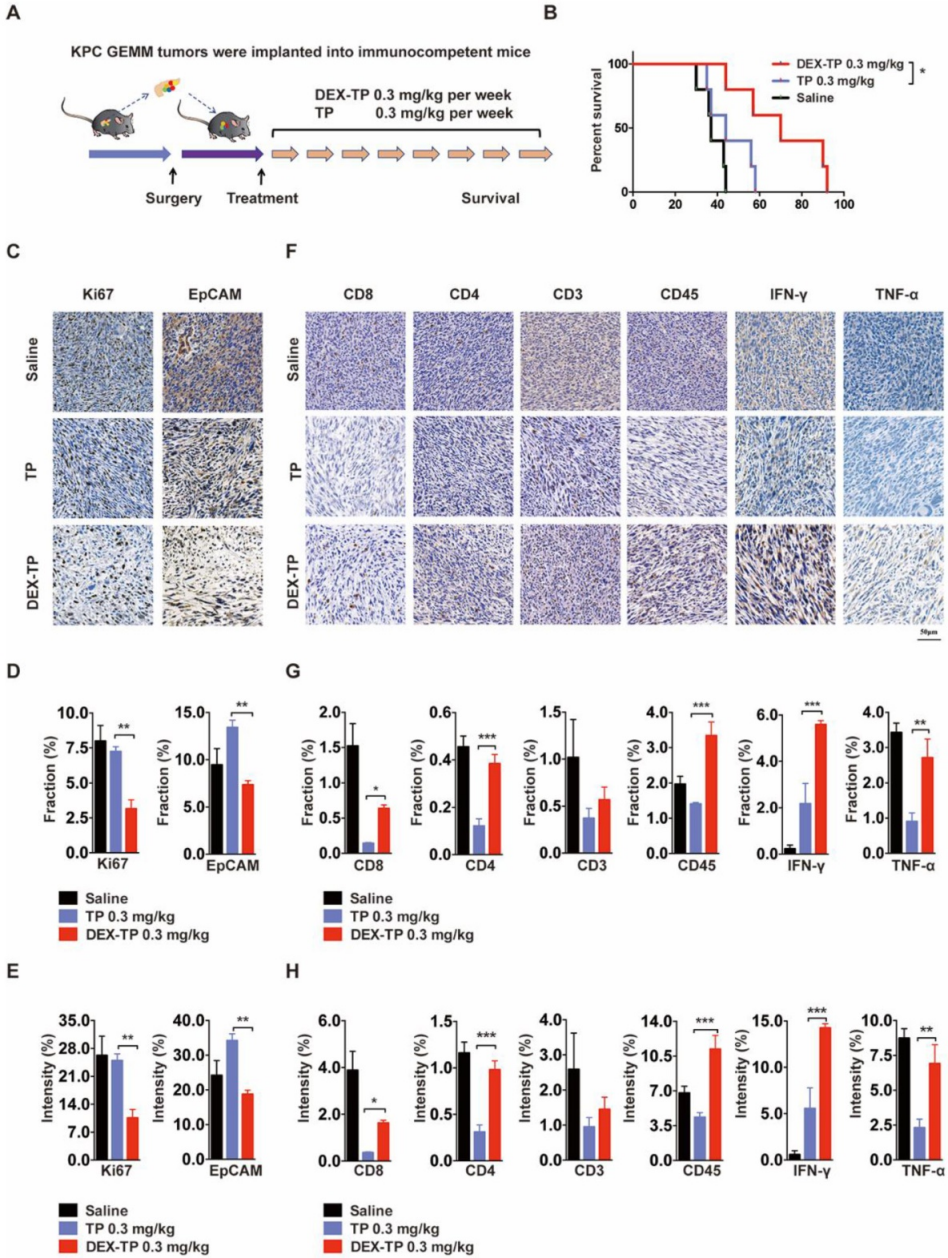
Therapeutic effects of DEX-TP in the KPC allograft orthotopic tumor model. (A) Scheme of the experiment. Treatments started 1 week after allograft surgery and were given weekly for a total of 8 treatments via tail vein injection with n=5 for all groups. (B) Kaplan-Meier survival curves of KPC tumor-bearing mice. (C, D and E) Ki67 (left) and EpCAM (right) analysis of tumor sections from each treatment group. Representative staining pictures (C), positive staining fraction (D) and staining intensity (E). Treatment groups from top to bottom: saline, TP 0.3 mg/kg, and DEX-TP 0.3 mg/kg. Doses are denoted in TP equivalent form. Error bars indicate the mean and SD, n=3. (F, G and H) Immune cell marker (CD8, CD4, CD3, and CD45) and effector cytokine (IFN-γ and TNF-α) analysis of tumor sections from each treatment group. Representative staining pictures (F), positive staining fraction (G) and staining intensity (H). Treatment groups from top to bottom: saline, TP 0.3 mg/kg, and DEX-TP 0.3 mg/kg. Doses are denoted in TP equivalent form. Error bars indicate the mean and SD, n=3.

We engaged an immunocompetent KPC allograft mouse model to investigate the effects of DEX-TP on tumor microenvironment. KPC allograft tumors share several clinically relevant pathology properties, such as marked fibrosis and a multicomponent composition 24, and to exclude variables, drugs were administered weekly here at equal doses **(Figure 5A)**. The overall survival after dextran-conjugated drug treatment was greater than that of free drug treatment **(Figure 5B)**, and the use of DEX-TP resulted in pronounced pancreas desmoplasia reduction, as suggested by the decreased Ki67 signature, wherein the proliferation of cancer cells was specifically hindered, as suggested by the decreased EpCAM signature **(Figure 5C, 5D and 5E)**. Additionally, the tumor immunity response to both treatments was quite different, as implied from the elevated expression levels of immune cell markers, including CD8, CD4, CD3 and CD45, as well as antitumor effector cytokines including IFN-γ and TNF-α. For free TP, apparent immunosuppressive phenotypes were observed, while for DEX-TP, such damage was apparently reversed **(Figure 5F, 5G and 5H)**.

## Discussion

PDAC is one of the most aggressive solid tumors worldwide, accounting for 2.5% of all cancer but 4.5% of all cancer related death 25. Surgical resection is the only chance for PDAC patients to be cured. However, due to late diagnosis, ~80% of them do not meet the criteria and rely heavily on drug treatments. Gemcitabine and 5-FU based chemotherapies remain the first-line therapy of PDAC, while approaches such as molecular targeting therapy and immunotherapy showed little progress despite various active investigations 26.

KRAS mutant cancer cells, being the progenitor of most pancreatic cancers 27, are defined by radical metabolic transformations to survive the hostile microenvironment, and treating them by exploiting such features has emerged as a fresh perspective that is worth trying 28. Our work here hijacking macropinocytosis with 70 kDa dextran provided a feasible KRAS targeting strategy from a drug delivery perspective. Dextran is a tunable material, a blood expander in the clinic, a PEG alternative in industry, and an endocytosis tracer in biological research 29. However, in previous reports, few attempts have been made to discuss its potential KRAS responsiveness as a carrier for drug delivery 12,30-32, which partially could be due to the underappreciated connection between oncogenic KRAS and macropinocytosis. Indeed, polymer-drug conjugates have been exploited for decades to improve drug circulation and tumor accumulation, benefiting from their high molecular weights. In this study, we found that KRAS-enhanced macropinocytosis could also contribute to an enhanced anti-tumor effect of the dextran conjugate compared to the free drug in KRAS mutant cancer. Dextran is chemically versatile to be tailored to conjugate with various cancer-targeting drugs, and smart linker chemistry design could further enhance tumor selectivity as needed.

The molecular weight or particle size dependent endocytic behavior has been observed previously, however, the underlying mechanism remains elusive. Dextran with different molecular weight could differ in particle size and degree of branching, both of which could affect their internalization. In fact, it was reported that higher molecular weight (i.e., 70 kDa) dextran could be more preferable compared with the lower molecular weight (i.e, 4 kDa) one^16^,^17^. It was speculated that macropinosomes might be preferentially labelled with larger particles over small ones. However, this could not explain the observation here that 150 kDa dextran didn't appear to be effectively engulfed through macropinocytosis, despite its large size. Furthermore, the oncogenic KRAS enhanced macropinocytosis reported here seems to suggest that the molecular size-dependency of dextran macropinocytosis is non-linear. Indeed, we tested another two dextran with Mw near 70 kDa: 20 kDa and 40 kDa dextran, but did not observe a similar macropinocytosis dependency and KRAS preference (**Figure S5**). Despite the fact that 70 kDa dextran was identified as the most suitable drug delivery carrier for KRAS targeting, there could be dextrans with other molecular sizes that could meet this purpose better. At the same time, the underlying molecular mechanism of macropinocytosis certainly remains further investigation.

We previously reported another KRAS targeting system, that is, the albumin system^33^. We considered the premise for albumin and dextran KRAS targeting to be quite different. Dextran enables a persistent cellular entry level difference between KRAS mutant cells and wild-type cells, while albumin undergoes distinct endosomal sorting but comparable cellular entry levels. Other delivery systems have been reported to benefit from KRAS mutation with enhanced intracellular processing 34-36, but one mechanistic understanding contribution of the dextran-aided case is that it signified that aberrant macropinocytosis activity may act as the single premise for KRAS targeting. A comparison of therapeutic merits among these systems could be interesting, although we are skeptical that it will be able to be ascertained.

TP is a powerful lead compound with novel pharmacological mechanism that can effectively restrain PDAC progression in a serial of pre-clinical models, however, whose clinical translation is greatly impaired by drug related severe toxicities. For instance, Kitzen *et al* disclosed that in a Phase I clinical trial of water-soluble TP derivative PG490-88, two patients died within 48 h after drug administration 3. Therefore, increasing TP delivery selectivity to cancer cells while reducing its toxicity to benign cells is a critical barrier for the clinical translation efforts of TP. While we have significantly improved the tolerability, selectivity and antitumor efficacy of TP using dextran conjugation in this study, its potential side effects on other macropinocytic cells still requires further investigation. Other potential risks of tissue injury, such as liver toxicity, etc., should also be throughout assessed considering the engulfing capability of hepatic macrophages 37.

Ultimately, we considered that the most encouraging result achieved by DEX-TP is its reduced collateral damage to the tumor immune microenvironment, since we believe that curative therapy for PDAC will ultimately be based on multicomponent combination therapy targeting the cancer described here, together with leveraging the immune system as well as other key components in the tumor microenvironment, such as fibroblasts. Therefore, a preserved immune system suggests higher clinical treatment value, and our platform presents a facilitating tool.

## Conclusion

Here, a cancer cell targeting strategy is provided for PDAC, engaging the demonstrated KRAS-responsive delivery carrier 70 kDa dextran. Dextran is a polymeric material with a long history of being a macropinocytosis marker; however, through a close look at the endocytosis mechanism and kinetics across different molecular weight dextrans under different KRAS mutation backgrounds, 70 kDa dextran was the only one recognized with KRAS selectivity among the tested sizes, as demonstrated by its enhanced cellular entry towards KRAS mutant cells compared to KRAS wild-type cells. The KRAS mutation sensitized cells with enhanced efficacy and cellular deposition efficiency towards the 70 kDa dextran-conjugated drug, and the conjugate constructed here, DEX-TP, not only demonstrated superior *in vivo* safety and efficacy but also further mitigated TP-induced immunosuppression within the tumors, adding greater value to its clinical applications. Thus, we repurposed macropinocytic dextran as a beneficial carrier for further applications for KRAS mutant PDAC therapy.

## Supplementary Material

Supplementary figures.

## Abbreviations

PDAC: pancreatic ductal adenocarcinoma
TME: tumor microenvironment
TP: triptolide
DEX-TP: 70 kDa dextran-conjugated TP
KPC: LSL-Kras^G12D/+^, LSL-Trp53^R172H/+^, Pdx-1-Cre
CLSM: confocal laser scanning microscopy
MTD: maximum tolerated dose
GEM: gemcitabine
