# Supplementary material for: Macropinocytic dextran facilitates KRAS-targeted delivery while reducing drug-induced tumor immunity depletion in pancreatic cancer
Source: Theranostics. 2022 Jan 1;12(3):1061–73. doi: 10.7150/thno.65299 (PMC8771557; doi:10.7150/thno.65299)
Supplement: Supplementary file 1 — Supplementary figures. [file thnov12p1061s1.pdf]

Supplementary Material

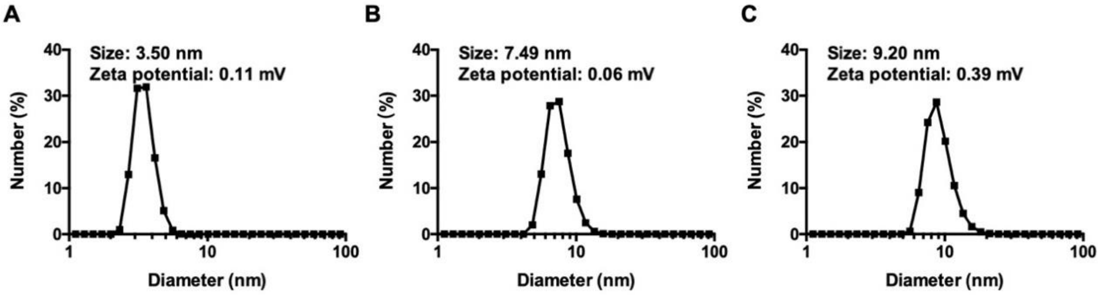

Supplementary Figure 1, Size distributions and zeta potentials of the (A) 4 kDa, (B) 70 kDa, and (C) 150 kDa dextrans.

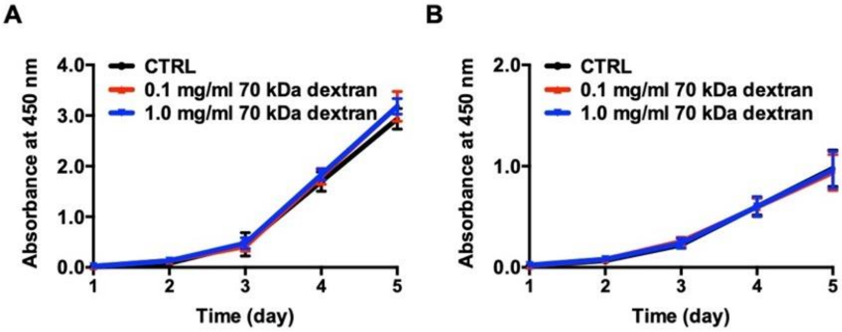

Supplementary Figure 2, Cell growth curves after 70 kDa dextran treatment. Tested cell lines: (A) MIA PaCa-2 and (B) BxPC-3. Error bars indicate the mean and SD, n=6.

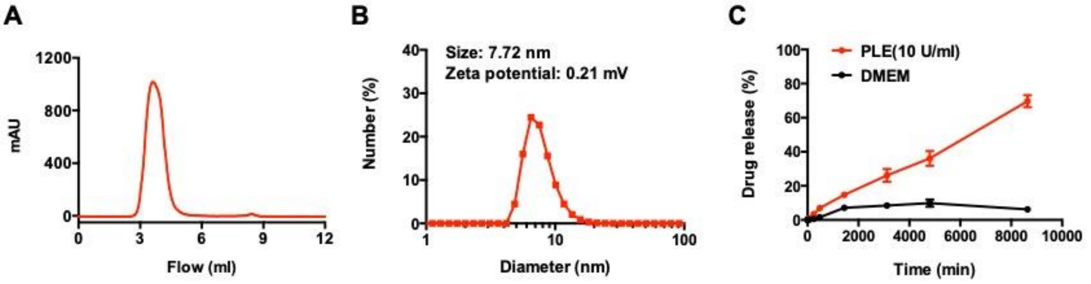

Supplementary Figure 3, Characterization of DEX-TP. (A) Desalting of TP succinate. The absorption was measured at 218 nm. (B) Size distribution and zeta potential of DEX-TP. (C) Drug release of DEX-TP in esterase-free DMEM solution (black) and 10 U/mL pig liver esterase (PLE)-containing medium. Error bars indicate the mean and SD, n=3.

Cell line		IC_{50} (TP)	IC_{50} (DEX-TP)	Deposition efficiency
KRAS mutant cells	MIA PaCa-2	10 nM	170 nM	5.9
	Panc-1	31 nM	389 nM	8.0
KRAS wild-type cells	HEK293T epithelial cells	5 nM	368 nM	1.4
	NIH3T3 fibroblasts	15 nM	7400 nM	0.2
	Jurkat T cells	5 nM	873 nM	0.6
	Raji B cells	8 nM	546 nM	1.5
KRAS manipulation	BxPC-3	12 nM	603 nM	2.0
	BxPC-3 $KRAS^{G12V}$	9 nM	123 nM	7.3

Max
Min

Supplementary Figure 4, IC_{50} values of TP and DEX-TP in different cell lines.

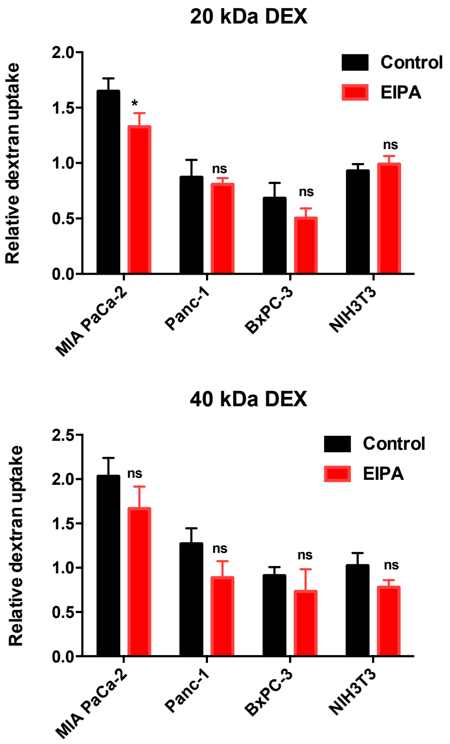

Supplementary Figure 5, Dextran uptake of 20 kDa (up) and 40 kDa (down) with (red) or without (black) 50 μM EIPA (a macropinocytosis inhibitor) in multiple cell lines. Data are presented relative to the values obtained for NIH3T3 cells without EIPA. KRAS mutant cell (MIA PaCa-2, Panc-1); KRAS wild-type cell (BxPC-3, NIH3T3). Error bars indicate the mean and SD, n=3.
